# MR-targeted TRUS prostate biopsy using local reference augmentation: initial experience

**DOI:** 10.1007/s11255-016-1283-2

**Published:** 2016-04-11

**Authors:** Wendy J. M. van de Ven, Wulphert Venderink, J. P. Michiel Sedelaar, Jeroen Veltman, Jelle O. Barentsz, Jurgen J. Fütterer, Erik B. Cornel, Henkjan J. Huisman

**Affiliations:** Department of Radiology and Nuclear Medicine, Radboud University Medical Center, P.O. Box 9101, 6500 HB Nijmegen, The Netherlands; Department of Urology, Radboud University Medical Center, Nijmegen, The Netherlands; Department of Radiology, ZGT, Hengelo, The Netherlands; Department of Urology, ZGT, Hengelo, The Netherlands

**Keywords:** MR–US fusion, Prostate cancer, Biopsy, PIRADS

## Abstract

**Purpose:**

To evaluate MR-targeted TRUS prostate biopsy using a novel local reference augmentation method.

**Patients and methods:**

Tracker-based MR–TRUS fusion was applied using local reference augmentation. In contrast to conventional whole gland fusion, local reference augmentation focuses the highest registration accuracy to the region surrounding the lesion to be biopsied. Pre-acquired multi-parametric MR images (mpMRI) were evaluated using PIRADS classification. T2-weighted MR images were imported on an ultrasound machine to allow for MR–TRUS fusion. Biopsies were targeted to the most suspicious lesion area identified on mpMRI. Each target was biopsied 1–5 times. For each biopsied lesion the diameter, PIRADS and Gleason scores, visibility during fusion, and representativeness were recorded.

**Results:**

Included were 23 consecutive patients with 25 MR suspicious lesions, of which 11 patients had a previous negative TRUS-guided biopsy and 12 were biopsy naïve. The cancer detection rate was 64 % (Gleason score ≥6). Biopsy was negative (i.e., no Gleason score) in seven patients confirmed by follow-up in all of them (up to 18 months). After MR–TRUS fusion, 88 % of the lesions could be visualized on TRUS. The cancer detection rate increases with increasing lesion size, being 73 % for lesions larger than 10 mm.

**Conclusion:**

Tracker-based MR–TRUS fusion biopsy with local reference augmentation is feasible, especially for lesions with an MR maximum diameter of at least 10 mm or PIRADS 5 lesions. If this is not the case, we recommend in-bore MR-guided biopsy.

## Introduction

An elevated or rising PSA followed by systematic (on average 12 core) transrectal ultrasound (TRUS)-guided biopsy (USgBx) is the currently internationally accepted diagnostic procedure to detect prostate cancer and determine patient management [1]. TRUS cannot localize malignant tissue and is merely used to guide systematic biopsies. USgBx has a low sensitivity (40 %) [2–4], causing three problems: (1) Significant cancers can be missed or underestimated; (2) there is unnecessary overtreatment due to overdiagnosis [5–7]; and (3) it may lead to repeat biopsies inducing increased infection rates [8]. Therefore, multi-parametric MR imaging and MR-guided biopsy might be a better alternative.

Multi-parametric MR imaging (mpMRI) has recently emerged as a diagnostic technique that can accurately localize significant cancer in the prostate [9, 10]. In-bore MR-guided MR biopsy (MRgMRBx) has been shown to (1) reduce the detection of low-risk cancer and (2) increase the detection rate of intermediate- and high-risk cancer, while using fewer cores [11]. However, the associated cost, relative complexity, and inconvenience of MRgMRBx may prevent widespread adaption in clinical practice. An alternative biopsy method for MR-guided biopsy would be welcome.

MR-guided TRUS fusion biopsy (MRgUSBx) has recently emerged [12–14]. This allows to combine the high accuracy of mpMRI with the ease and accessibility of TRUS. However, for 95 % correct Gleason grading, a 1.9-mm accurate spatial registration of MR and US is required [15]. Most MRgUSBx devices do not achieve this accuracy in practice (3–6 mm [12, 16]). Accuracy can be slightly increased by taking one or more additional cores [17].

Two MRgUSBx strategies can be distinguished: cognitive and computational fusion. The fastest and simplest form of computational fusion is tracker-based rigid registration, using an electromagnetic (EM)-tracker [18, 19]. An EM-tracker attached to a TRUS probe tracks its position and orientation allowing to link a live TRUS image to a prerecorded MR image. We previously performed a phantom study on EM-tracker registration and estimated the registration accuracy in 3D to be 5–7 mm [20]. Current rigid MR–TRUS fusion protocols focus on optimizing accuracy for the entire gland volume. Due to prostate deformation, the registration accuracy can never be optimal within the whole gland. We hypothesize that by restricting the registration to the partial gland volume surrounding the lesion, a more consistent and possibly better registration accuracy can be achieved within this partial volume containing the lesion. The EM-tracker approach we use in our study allows to do this quickly, which, combined with visual feedback, can lead in a few iterations to an augmented, focal match of TRUS and MR imaging. We propose a novel protocol to augment the accuracy locally by selecting reference landmarks on both MR and TRUS images that are close to the biopsy target [21], which we refer to as local reference augmentation in analogy to all-weather aircraft landing systems.

The aim of this study is to evaluate our novel EM-tracker MR–TRUS fusion biopsy protocol using local reference augmentation in regular clinical practice. To our knowledge, this is the first report on a locally optimized MR–TRUS fusion biopsy method. We will explore the capability of sampling mpMRI suspicious lesions and get insight into the representativeness of the biopsy result. Additionally, we will determine the proportion of tumors confidently visible on TRUS after fusion.

## Patients and methods

### Patient population

Inclusion criteria for our study were patients scheduled for MRgUSBx who had an mpMRI showing a lesion scored as PIRADS ≥4 or PIRADS 3 with additional clinical suspicion (e.g., unusually high PSA, persistent rising PSA). Biopsy was performed at the Radboudumc (Nijmegen, the Netherlands) or at the ZGT (Hengelo, the Netherlands). The study was approved by the Institutional Review Board of the Radboudumc for MR lesions >9 mm, and all included Radboudumc patients gave their written informed consent. The requirement to obtain institutional review board approval was waived at ZGT as MRgUSBx was their regular clinical procedure in prostate cancer diagnosis. Our data set contains all patients included at the Radboudumc and ZGT between September 2013 and October 2014.

### Multi-parametric magnetic resonance imaging

Prostate imaging mpMRI sequences were compliant to the ESUR guidelines [22] and included three orthogonal T2-weighted, diffusion-weighted, and dynamic contrast-enhanced (DCE) series. Apparent diffusion coefficient (ADC) maps were calculated by the scanner. DCE used a gadolinium-based contrast agent by injecting 15 mL of Dotarem intravenously. Preferentially, we also added a 3D T2-weighted sequence with an isotropic resolution of 1 mm for MRgUSBx. Images of the entire prostate gland and seminal vesicles were obtained using a 3 Tesla MRI scanner (MAGNETOM Trio or Skyra; Siemens, Erlangen, Germany) with either a pelvic phased-array coil or a combination of an endorectal and pelvic phased-array coil.

Several genitourinary radiologists experienced in prostate MRI prospectively evaluated the mpMRI in a regular clinical setting, using structured reporting with the ESUR-standardized PIRADS classification [22]. The location of each lesion was stored on an in-house-developed mpMRI analysis, viewing and reporting workstation (ProCAD) [23]. All mpMRI evaluations were discussed in a consensus meeting and adapted if necessary.

### Biopsy procedure

An Aplio 500 (Toshiba Medical Systems, Japan) ultrasound device with an end-firing transrectal transducer (PVT-781VT; Toshiba Medical Systems, Japan) was used for the MRgUSBx. Previously obtained T2-weighted MR images were uploaded to the ultrasound device. The original mpMRI including PIRADS scores were displayed on our mpMRI workstation, available during the fusion procedure. For a peripheral zone lesion, the biopsy target was the darkest lesion region on ADC; for the transition zone, it was the most suspicious area on the T2-weighted series. The MR target location was first identified on the workstation displaying the mpMRI and then re-located on the uploaded T2-weighted image.

**Fig. 1:**
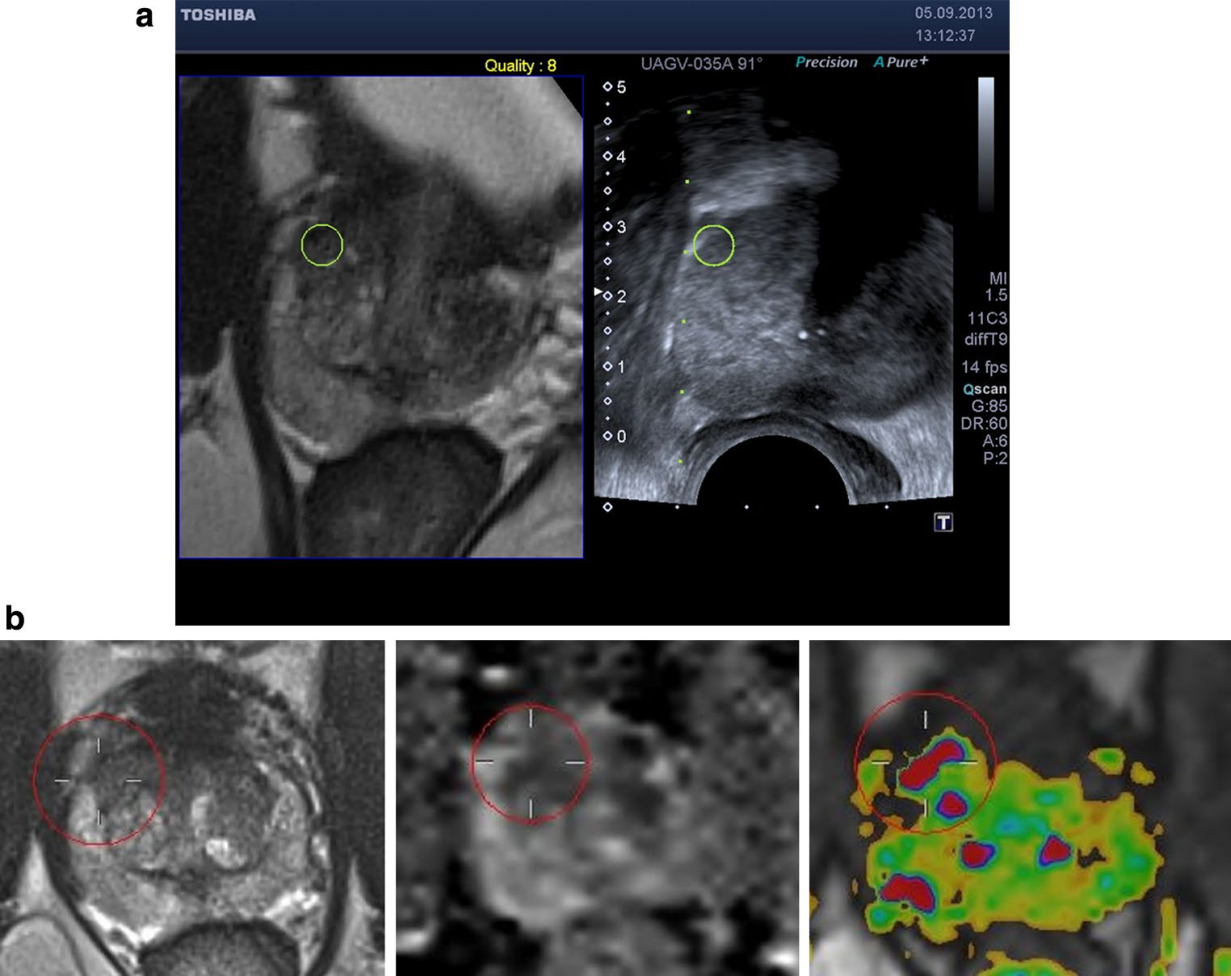
**a** Screenshot of the Toshiba Aplio 500 during MRgUSBx. The *green circle* indicates the target as reported on mpMRI, projected on the US image after fusion. The *dotted green line* indicates trajectory along which the needle will shoot in the prostate (to be moved slightly for correct targeting in this screenshot). **b** The corresponding mpMRI with from *left* to *right* the transversal T2-weighted image, ADC map, and DCE image. These images were displayed using ProCAD and were available during the fusion procedure

**Fig. 2:**
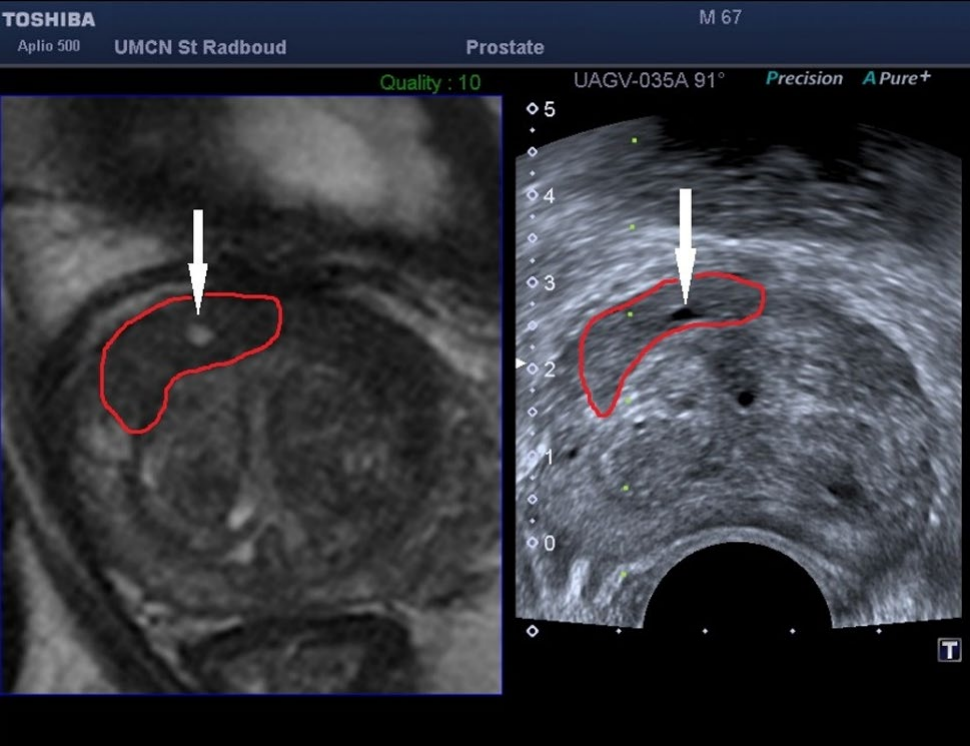
Screenshot of the Toshiba Aplio 500 demonstrating the use of anatomical landmarks used for local reference augmentation. Reference landmarks are (iteratively) selected close to the target location. This example shows a cyst (indicated by the *white arrow*) inside the lesion (segmented in *red*)

Patients were positioned in the left lateral position for biopsy, similar to USgBx. The TRUS probe was inserted rectally with gel. A needle guide was placed onto the transducer. The ultrasound machine had a SmartFusion option that includes an EM position sensor attached to the TRUS transducer to spatially correlate imported 3D MR images and US in real time. The SmartFusion EM-tracker-based fusion is a two-step process. First, the US scanning orientation is matched to a variable MR image-reformatted orientation by manually selecting the best matching reformatting angle. Secondly, the correct anatomical 3D position is linked by selecting the same reference anatomical landmark in both images. During the biopsy procedure, the live US and the pre-acquired transversal T2-weighted images were shown simultaneously, allowing MR image guidance (example shown in Fig. 1). The accuracy of the EM-based method was enhanced by our novel local reference augmentation, i.e., the reference landmark used for synchronizing 3D position was selected close to the target location as identified on MR (example shown in Fig. 2). The MR-identified lesion location is then re-located on ultrasound using the fused image display. Visible mismatch was minimized by repeating the landmark selection, compensating for landmark localization errors or patient movement. In case the lesion was visible on TRUS after initial fusion, the biopsy was targeted to the TRUS location. Note that visibility in this respect means that lesions become visible on ultrasound only during the MR targeting fusion procedure. They are much less visible on ultrasound as such without the aid of fusion. Each mpMRI-detected target was biopsied 1–5 times. MR–TRUS fusion screenshots were stored during the biopsy to record the exact needle core location as part of the procedure to assess the representativeness.

### Histopathology

Similar to all prostate biopsy procedures, all biopsy core specimens were examined by one of two specialized urogenital pathologists and graded according to the 2005 International Society of Urological Pathology Modified Gleason Grading System [24]. For stratification of biopsy data into significant and insignificant cancer, we applied the criteria for MRgMRBx as published by Pokorny et al. [11]. In short, lesions with a Gleason score ≥7 in at least one of the MRgUSBx cores were defined as being clinically significant, as well as high-volume Gleason 3+3 (i.e., tumor length >6 mm or more than 2 positive cores). Low-volume Gleason 3+3 or Gleason scores <6 were considered clinically insignificant, and lesions for which no Gleason score could be determined were considered negative. The cancer detection rate was based on lesions with Gleason score ≥6. The pathology results were correlated with the MR–TRUS fusion biopsy images and the original mpMRI study.

### Biopsy evaluation

A urologist in consultation with a radiologist evaluated the pathological outcome of the biopsy, taking the PIRADS scores of the mpMRI into consideration. In case the pathological outcome is lower than what would be expected based on mpMRI, the patient would need a re-biopsy. The biopsy was considered representative if the patient was not scheduled for an immediate MRgMRBx re-biopsy. As part of the radiological quality control procedure, all mpMRI studies that did not have significant cancer after MRgUSBx were re-evaluated by two expert prostate radiologists in consensus. Furthermore, follow-up results of patients were collected.

### Data analysis

As an indication of feasibility, the cancer detection rate and number of non-representative biopsies were analyzed. Earlier research indicated that PIRADS and Gleason score, as well as visibility and lesion size, had an effect upon the detection rate, and therefore we also performed subgroup analyses. Statistical proportion analysis was performed to determine the cancer detection rate for each (sub)group. Three different size groups were created (0–10, 11–20, and ≥20 mm). Finally, the mean number of cores taken per lesion was determined for each size group.

## Results

Between September 2013 and October 2014, 23 consecutive patients with 25 mpMRI suspicious lesions underwent MRgUSBx and were included (two patients had two lesions). Table 1 summarizes the general characteristics of the included patients. Eleven patients had at least one previous negative TRUS biopsy session, one patient had a previous negative MRgMRBx 1 week before MRgUSBx (but representativeness of MRgMRBx was uncertain), and the other 11 patients were biopsy naïve. The prospective mpMRI scores were: 3 PIRADS 3 lesions, 9 PIRADS 4 lesions, and 13 PIRADS 5 lesions. Most of the mpMRI suspicious lesions were located in the peripheral zone (20/25).

**Table 1:** Summary of patient characteristics

Parameter	All patients, *n* = 23			
	Mean	SD	Median	Range
Age (years)	63	6.4	65	51–75
PSA (ng/mL)	10.3	6.2	8.9	2.9–29.3

All MRgUSBx were considered representative, none needed an immediate re-biopsy. Table 2 shows the results of the prostate biopsies. In summary, the median number of targeted cores taken per lesion was 2 (range 1–5). Cancer (Gleason score ≥6) was detected in 16 of the 25 lesions (64 %) and 16 of 23 patients (70 %). Two patients had a second lesion with Gleason score ≥6. MRgUSBx was negative in 7 patients: 2 had a PIRADS 5 lesion, 4 had a PIRADS 4 lesion, and 1 had a PIRADS 3 lesion. Patients with negative MRgUSBx were referred to active surveillance based on PSA or follow-up mpMRI after 3–6 months.

**Table 2:** Results of prostate biopsies

Parameter	Value
No. of patients/lesions	23/25
No. of patients/lesions with cancer (GS ≥6)	16/16
Total no. of cores	64
No. of positive cores (GS ≥6)	28
Mean primary Gleason grade	3.19 ± 0.39
Mean secondary Gleason grade	3.25 ± 0.43
Mean Gleason score (GS)	6.43 ± 0.50
No. of GS 3+3	9 (of which 4 were clinically significant)
No. of GS 3+4	4
No. of GS 4+3	3

**Fig. 3:**
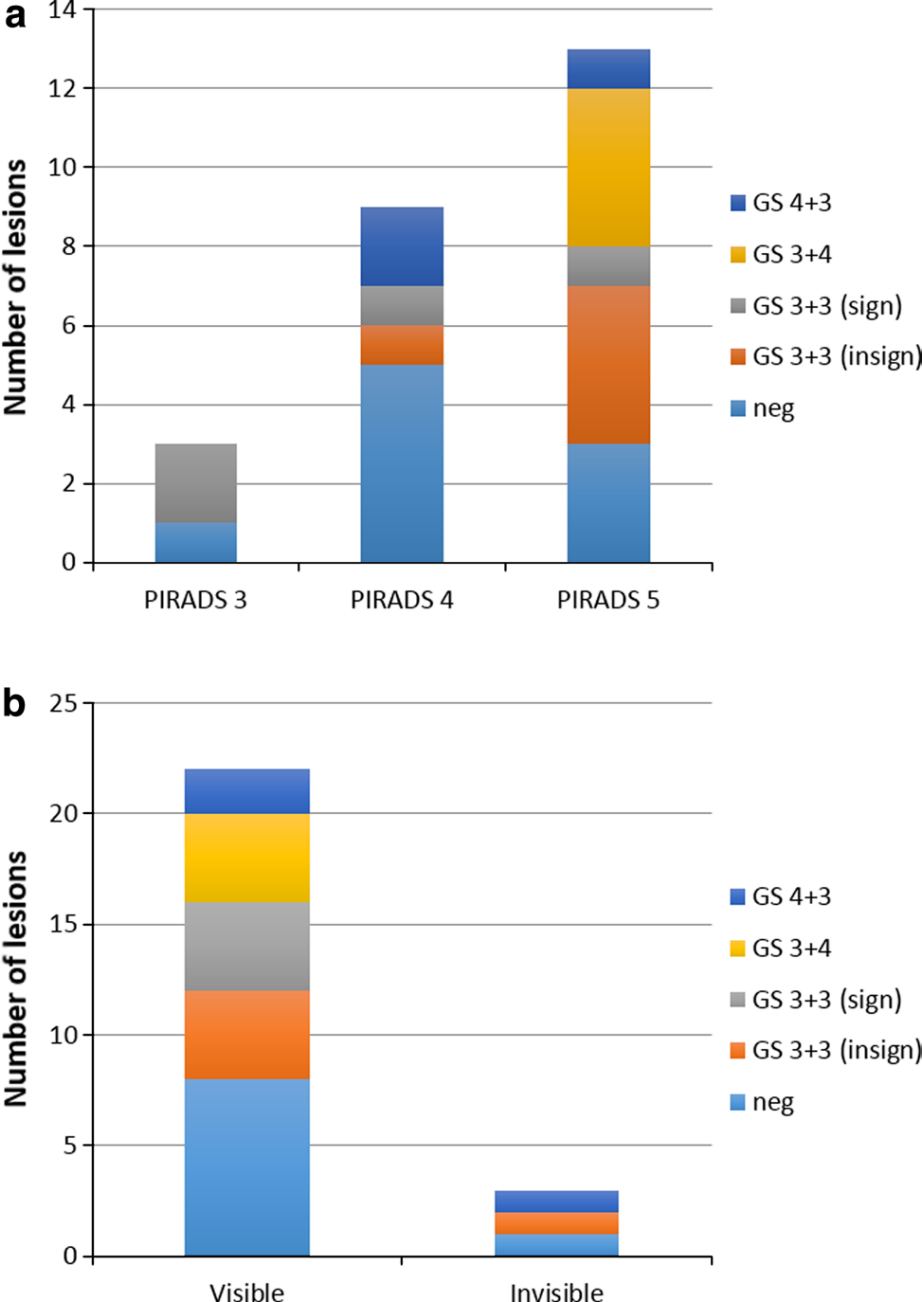
The number of lesions detected with targeted MRgUSBx according to **a** the PIRADS score on mpMRI and **b** the visibility of the lesion on TRUS

The cancer detection rates per PIRADS score are shown in Table 3. The cancer detection rate of the PIRADS 5 lesions was 77 %, of PIRADS 4 lesions 44 %, and of the PIRADS 3 lesions 67 %. Pathological biopsy outcomes per PIRADS score are shown in Fig. 3a. Clinically significant lesions were present in 46, 33, and 67 % of the PIRADS 5, 4, and 3 lesions, respectively.

**Table 3:** Cancer detection rates per PIRADS score and TRUS lesion visibility, including 95 % confidence intervals (CI)

Category	No. of lesions	No. of lesions with any cancer	Proportion (95 % CI) any cancer	No. of lesions with significant cancer	Proportion (95 % CI) significant cancer
PIRADS 3	3	2	67 % (20–94 %)	2	67 % (20–94 %)
PIRADS 4	9	4	44 % (19–73 %)	3	33 % (12–65 %)
PIRADS 5	13	10	77 % (49–93 %)	6	46 % (23–71 %)
TRUS visible	22	14	64 % (43–80 %)	10	45 % (27–65 %)
TRUS invisible	3	2	67 % (20–94 %)	1	33 % (6–80 %)
All	25	16	64 % (44–80 %)	11	44 % (27–68 %)

During the biopsy procedure, 23 of the lesions (88 %) could be visualized on TRUS after image registration. For TRUS visible lesions, biopsies were targeted to the location as visible on TRUS. The cancer detection rate of the TRUS visible lesions was 64 % and that of the TRUS invisible lesions 67 % (see Table 3). For clinically significant cancer, this changes to 45 and 33 % for TRUS visible and invisible lesions, respectively. The pathological outcomes for TRUS visibility are shown in Fig. 3b.

In Table 4 the lesions are grouped according to their size. The cancer detection rate is higher for the larger lesions, on both lesion and core basis. For lesions larger than 10 mm, the cancer detection rate is 73 % (Gleason score ≥6). Also, slightly more cores are taken for larger lesions.

**Table 4:** Biopsy outcomes grouped according to lesion size on mpMRI

Largest diameter (mm)	No. of lesions	Mean no. of cores (range)	Cancer (GS ≥6) detection rate per lesion (95 % CI)	Cancer (GS ≥6) detection rate per core (95 % CI)
0–10	10	2.4 (2–3)	50 % (24–76 %)	33 % (18–53 %)
11–20	11	2.5 (1–5)	64 % (35–85 %)	44 % (28–63 %)
>20	4	2.8 (2–4)	100 % (45–100 %)	73 % (43–91 %)

The follow-up of the mpMRI suspicious patients with a negative or insignificant MRgUSBx outcome was collected, and results are shown in Table 5. In summary, follow-up results showed that 3 of 7 patients with negative MRgUSBx had stable or decreasing PSA after 6–18 months and one patient is in active surveillance. For the remaining three, one had negative USgBx after 12 months, one had negative MRgMRBx after 12 months, and one showed a PIRADS 2 lesion on follow-up mpMRI. For the five patients with insignificant MRgUSBx, one patient was lost to follow-up, one patient has decreasing PSA, one patient is scheduled for biopsy, and two are still in active surveillance.

**Table 5:** Re-evaluation of original mpMRI and follow-up results for negative and clinically insignificant GS 3+3 outcomes

Biopsy outcome	PSA (ng/mL)	Original PIRADS	PZ/TZ	Re-evaluated PIRADS	Re-evaluation comments	Follow-up results
Negative						
	23	5	PZ	2^a^	Negative MRgMRBx 1 month earlier	Still in active surveillance
	16	5	PZ	5	Biopsy may not have been representative	Stable PSA after 6 months and mpMRI is unchanged
	6	4	TZ	4	Biopsy results were considered acceptable during re-evaluation of mpMRI	Increasing PSA to 8.4 and now PIRADS 5 lesion on mpMRI after 12 months; MRgMRBx was negative
	9.7	4	PZ	4	Biopsy may not have been representative	Stable PSA after 6 months
	3.9	4	PZ	4	Negative biopsy outcome is acceptable	PSA increased to 5.1, but USgBx was negative after 12 months
	6.1	4	TZ	3^a^	Biopsy outcome prostatitis is acceptable	PSA decreased to 3.9 after 6 months
	7.3	3	PZ	2^a^	Negative biopsy outcome is acceptable	After 12 months, mpMRI showed a PIRADS 2 lesion corresponding to prostatitis
GS 3+3						
	13	5	PZ	4^a^	Biopsy was representative	Lost to follow-up
	6.4	5	PZ	5	Tumor volume in biopsy is small regarding the significant tumor visible on mpMRI	PSA increased to 7.7 after 18 months, patient in active surveillance
	9	4	PZ/TZ	3^a^	Biopsy was representative	Decreasing PSA to 8.1 after 12 months
GS 3+3 and negative (patients with more than one lesion)						
	11	5	PZ	5	Biopsy was representative	Patient scheduled for biopsy
		5	PZ	5	Biopsy may not have been representative, difficult location to target	
	6.3	5	PZ	4^a^	Biopsy was representative	Still in active surveillance
		4	PZ	4	Small lesion, biopsy outcome may not have been representative	

*PZ* peripheral zone, *TZ* transition zone

^a^Lesions that have been downgraded in re-evaluation of the original mpMRI

## Discussion

Prostate cancer (Gleason score ≥6) was detected in 64 % of the lesions biopsied and in 70 % of the patients. All biopsies were representative showing that our novel local reference augmented method is feasible in clinical practice. During MR–TRUS fusion, 88 % of the lesions could be visualized on TRUS alone, allowing targeted biopsies to be optimized using live TRUS guidance. The cancer detection rate increases with increasing tumor size.

The representativeness of the mpMRI suspicious, but negative MRgUSBx was confirmed by follow-up; i.e., none of the seven negative MRgUSBx patients revealed clinically significant pathology. The detection rate for clinically significant cancer (44 %) was lower than the 65 % shown by Pokorny et al. [11] for MRgMRBx. Three reasons can be pointed out for this difference: (1) different patient population between studies; (2) difference in biopsy technique; and (3) difference in expertise of the radiologist(s). In Pokorny et al. three expert radiologists in consensus evaluated the mpMRI, which in our study was done by the attending prostate radiologist. To investigate the first two points, more research is required comparing MRgUSBx to MRgMRBx with similar patient populations. To investigate the third point, two expert radiologists in consensus re-evaluated the original mpMRIs with negative and clinically insignificant MRgUSBx. Table 5 shows that the original mpMRI assessment may indeed have overestimated tumor aggression: 43 % (6/14) of the lesions were downgraded during retrospective re-evaluation by experts. This confirms that expertise is important. In case subsequent biopsy reveals no clinically significant cancer in PIRADS 4 and 5 lesions, it is very important to re-evaluate the quality of the mpMRI, the reading, and the subsequent biopsy technique.

The original results of our locally optimized EM-based registration method are well in line with results of other EM-based systems, which have a cancer detection rate between 49 and 69 % [18, 19]. Clinical studies with other MR–TRUS fusion systems also show similar detection rates [18, 19, 25, 26]. However, patient populations differ quite a bit between the different studies, e.g., regarding the amount of patients with a previous negative biopsy or patients that were biopsy naïve. Although our initial results are similar to previously published results, more research is needed to investigate whether local reference augmentation is an actual technical improvement to current MR–TRUS fusion methods.

We know from previous phantom studies that our EM-based registration method has a registration accuracy of about 5 mm [20]. Yet we were still able to achieve reasonable detection rates in the 0–10 mm category. The following items played a role. First, we enhanced the EM-based registration technique by locally optimizing fusion through the iterative selection of anatomical landmarks close to the target. Secondly, the number of biopsy cores was increased in case the performing physician was not certain about the biopsy taken. By taking more cores per target, the tumor hit rate increases [17]. Thirdly, during the MRgUSBx we noticed that 88 % of the targets became visible on TRUS images during MR–TRUS fusion. In case the targets become visible on TRUS, these can be more accurately targeted even if the registration is not optimal [27]. The TRUS visibility is often subtle and may very well depend on the quality of the ultrasound images.

The cancer detection rate increases with increasing lesion size, which might be an indication that the smaller lesions are harder to hit with MRgUSBx and might better be biopsied with MRgMRBx. For lesions larger than 10 mm diameter, our results show a cancer detection rate of 73 %, approaching the results of MRgMRBx.

The main limitation of our study is the small number of patients. But sufficient to indicate that MR–TRUS fusion with local reference augmentation is feasible for targeting prostate biopsies. To investigate whether MRgUSBx is a viable alternative to MRgMRBx, a non-inferiority trial setting including more patients is required. Then, similar patient groups can be compared and it can be determined whether MRgUSBx is non-inferior to MRgMRBx.

To summarize, MR–TRUS fusion biopsy using local reference augmentation is feasible. This is especially the case for lesions with an mpMRI maximum diameter of at least 10 mm or PIRADS 5 lesions. Smaller lesions may still require in-bore MR-guided biopsy.
